# Venereal Prevention

**Published:** 1920-07-17

**Authors:** 


### THE PUBLIC WELL-BEING.

### Venereal Prevention.

### A Layman's Views.

*[We have received the following letter from a correspondent, who signs himself "Social Reformer." We print it as it stands, and will welcome correspondence on the subject.]*

I agree with the leading article on venereal prevention in last week's issue of THE HOSPITAL to this extent—if I understand it aright—that the complex problem of venereal disease is not likely to be settled, or even greatly modified, by the single proposal put before the Minister of Health that the Government and local authorities should "instruct" all qualified chemists "to sell such means of immediate self-disinfection . . . as may be approved from time to time by the Ministry of Health or by medical officers of health." But I hold greater respect for Dr. Addison's consideration of public opinion than the leading article seems to imply, and, in view of all the circumstances, I feel that caution is necessary.

Two considerations arise upon the proposal put before the Minister. The first is how far either the Ministry or local authorities will be able to impose their will upon qualified chemists, and therefore how far the latter may be expected to carry out any "instructions" as far as they are intended to go; the second is the seriously conflicting opinions, both in the professional mind and in the public mind, with regard to the wisdom and propriety of placing within easy and immediate reach of young people of both sexes the means of performing acts of immorality with comparative impunity. The conviction is strongly held by a large number of sociologists and others who have given anxious attention to this subject that the remedy may be far worse than the disease by encouraging vice and steeping it in a false sense of security.

This point of view was appreciated to its fullest extent by Dr. Addison in his reply to the deputation. He admitted that "a great wave of public feeling" would probably be antagonistic to an official and deliberate policy, not only of teaching the means of immediate self-disinfection, but of making those means available as a matter of course. As far as can be gathered, he is dubious of success even if such a policy could be thoroughly and scientifically applied. But could it be so applied? A strong point has been made of the fact that these methods were employed successfully, at least during the later stages of the war, in the British Army. But it is easy to exaggerate the significance and ultimate value of the system created by the military authorities. It is perfectly clear, as Dr. Addison indicated, that it is impossible to deal with a population of more than 40,000,000 civilians on the same basis and with the same scrupulosity that might seem to be easy in the case of an army under the strictest discipline and under the watchful supervision of an efficient medical corps with full powers of inspection and in close daily touch with the troops. Further than that, however, the success of the measures used among the soldiers was by no means as complete as is commonly reported. Last year, it will be remembered, Dr. Addison appointed a strong body of men to investigate the subject, and they reported to him that certain drugs, if properly applied, were efficacious; but if they were not properly and scientifically applied their efficacy could not be relied upon. They also reported that the use of such drugs encouraged the taking of risks, and that, "in spite of the most careful instructions, the issue of packets to soldiers resulted in men who used them for self-disinfection still having infection." This conclusion appears to be borne out in some measure by the reports from many hospitals throughout the country that a large percentage in the increase of venereal disease is provided by ex-soldiers.

It is evident, therefore, that public-spirited and organised efforts to deal with this dangerous scourge of humanity must proceed with caution, and that whatever general policy is ultimately adopted must be derived from a comprehensive knowledge of the psychological factors in the situation, as well as from scientifically prepared data that can be regarded as completely reliable. Such experiments as are being carried on at Manchester and elsewhere will be watched with anxious interest, and may afford valuable assistance in the final solution. Meanwhile, it is all to the good that such organisations as the Society for the Prevention of Venereal Disease are carrying on a wide and persistent campaign of instruction and enlightenment as to the causes and deadly consequences of the disease.

It is also to the good that the medical and the lay Press should keep the problem prominently and sensibly before the public. I thoroughly appreciate your impatience, but a false step at this moment might have disastrous consequences. I should very likely be prepared to go further than you in the end, but not so fast.

